# Quantification of Liver Fat Content after Radiofrequency Ablation for Liver Cancer: Correlation with Hepatic Perfusion Disorders

**DOI:** 10.3390/diagnostics11112137

**Published:** 2021-11-18

**Authors:** Li-Shan Shen, Quan-Xi Li, Xiao-Wen Luo, Hui-Jun Tang, You-Jie Tang, Wen-Jie Tang, Ruo-Mi Guo

**Affiliations:** Department of Radiology, The Third Affiliated Hospital, Sun Yat-Sen University, Guangzhou 510635, China; shenlsh3@mail2.sysu.edu.cn (L.-S.S.); liquanxi@mail.sysu.edu.cn (Q.-X.L.); wendy_8262@163.com (X.-W.L.); tanghj7@mail.sysu.edu.cn (H.-J.T.); tangyj35@mail.sysu.edu.cn (Y.-J.T.)

**Keywords:** fatty liver, hepatic perfusion disorders, magnetic resonance imaging, fat quantification

## Abstract

Purpose: To quantitatively investigate the correlation between liver fat content and hepatic perfusion disorders (HPD) after radiofrequency ablation (RFA) for liver cancer using magnetic resonance imaging (MRI)-determined proton density fat fraction (PDFF). Materials and methods: A total of 150 liver cancer patients underwent liver MRI examination within one month after RFA and at four months after RFA. According to the liver fat content, they were divided into non-, mild, moderate, and severe fatty liver groups. The liver fat content and hepatic perfusion disorders were determined using PDFF images and dynamic contrast-enhanced MRI images. The relationship between the liver fat content and HPD was investigated. Results: At the first postoperative MRI examination, the proportion of patients in the nonfatty liver group with hyperperfused foci (11.11%) was significantly lower than that in the mild (30.00%), moderate (42.86%), and severe fatty liver (56.67%) groups (*p* < 0.05), whereas the proportions of patients with hypoperfused foci (6.67%, 7.5%, 5.71%, and 6.67%, respectively) were not significantly different among the four groups (*p* > 0.05). In the nonfatty liver group, the liver fat content was not correlated with hyperperfusion abnormalities or hypoperfusion abnormalities. By contrast, in the three fatty liver groups, the liver fat content was correlated with hyperperfusion abnormalities but was not correlated with hypoperfusion abnormalities. At the second postoperative MRI examination, six patients in the nonfatty liver group were diagnosed with fatty liver, including two patients with newly developed hyperperfusion abnormalities and one patient whose hypoperfusion abnormality remained the same as it was in the first postoperative MRI examination. Conclusion: There was a high correlation between the liver fat content and hyperperfusion abnormalities after RFA for liver cancer. The higher the liver fat content was, the higher the was risk of hyperperfusion abnormalities. However, there was little correlation between liver fat content and hypoperfusion abnormalities, and the increase in postoperative liver fat content did not induce or alter the presence of hypoperfused foci.

## 1. Introduction

Hepatic perfusion disorders (HPD) are transient differences in the degree of hepatic enhancement, which are differences in blood perfusion among hepatic segments, subsegments, and lobes caused by various factors [1,2,3]. The in-depth study of HPD has reported that abnormal liver fat deposition associated with fatty liver, which causes hepatocyte swelling and hepatic sinusoidal compression [4], affects the degree of blood perfusion, which manifests as HPD during contrast-enhanced imaging [5]. However, it has not been reported whether the increase in liver fat deposition aggravates HPD or what changes in hyperperfusion and hypoperfusion abnormalities occur when liver fat deposition increases. Patients who have fatty liver after radiofrequency ablation (RFA) for liver cancer often show abnormal perfusion foci during contrast-enhanced imaging related to the portal vein injury or compression [6]. It is always necessary and an issue to distinguish recurrent tumors from benign transient perfusion anomalies.

Conventional imaging examinations, such as ultrasound, computed tomography (CT), and magnetic resonance imaging (MRI), can accurately diagnose the presence or absence of fat deposition in the liver but cannot accurately quantify the fat content in the liver [7,8] and cannot accurately determine whether the fat content in the liver is associated with HPD. With the development of imaging technology, a noninvasive quantitative MRI technique called proton density fat fraction (PDFF) can noninvasively and quantitatively measure the fat content in organs, evaluate lipid metabolism, provide information on the fat spatial distribution [9,10], and easily and rapidly calculate the fat fraction in the liver [11,12,13]. In this study, we quantitatively measured the liver fat content in patients after RFA for liver cancer using the PDFF and analyzed HPD to investigate the relationship between the liver fat content and HPD.

## 2. Materials and Methods

### 2.1. Clinical Data

**Figure 1:**
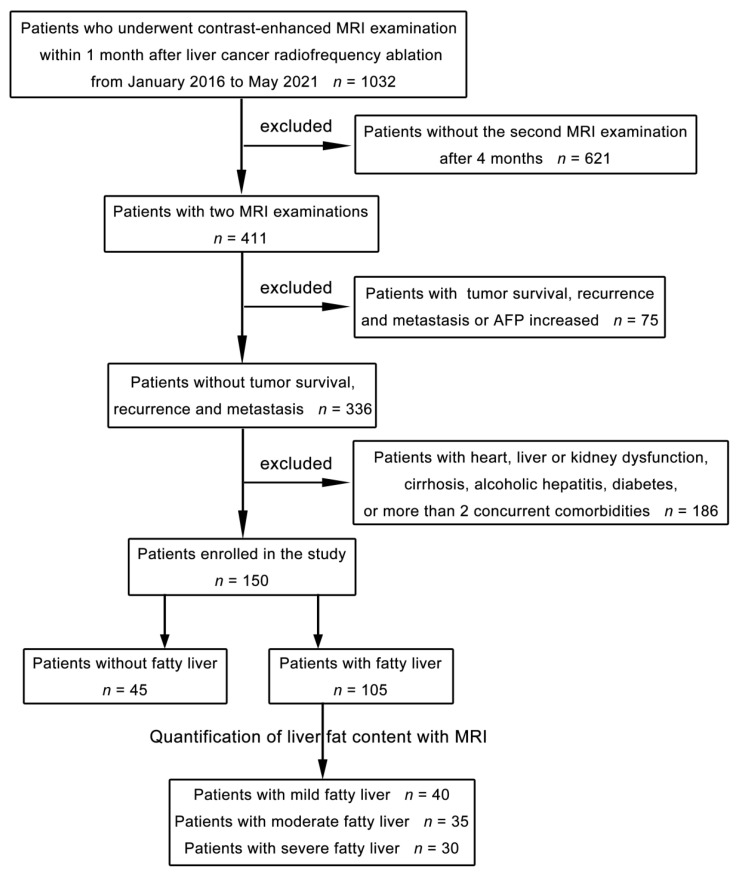
Flowchart of the patient selection process.

A total of 150 patients who underwent RFA for liver cancer at our hospital between January 2016 and May 2021 were acquired retrospectively in this study. All patients were diagnosed with small hepatocellular carcinoma (diameter ≤ 3 cm) without vascular or bile duct invasion. None of them had lymph node or distant metastasis. The patients were divided into a nonfatty liver group and a fatty liver group according to the presence or absence of fatty liver in MRI examination with PDFF (IDEAL-IQ sequence, GE Healthcare) before RFA. There were 45 patients in the nonfatty liver group, including 25 males and 20 females, with an age range of 35–64 years and a mean age of 51.5 years. There were 105 patients in the fatty liver group, including 65 males and 40 females, with an age range of 34–67 years and a mean age of 50.5 years. The patient selection flowchart is shown in Figure 1. No signs of tumor survival, recurrence or metastasis were found in any of the patients after RFA. Postoperative serum alpha-fetoprotein was normal in all patients. No recurrence was found in one-year follow-up ultrasound examination. Patients with an allergy to contrast agents, insufficiency of the heart, liver, or kidney, cirrhosis, alcoholic hepatitis, diabetes mellitus, or more than two concurrent comorbidities were excluded (Table 1). The first postoperative contrast-enhanced MRI examination with PDFF of the liver was performed within one month after the RFA, and the second postoperative contrast-enhanced MRI examination with PDFF of the liver was performed four months after the RFA. The study was approved by the local research and ethics committee, and all patients gave their informed consent prior to their inclusion in this study.

### 2.2. Equipment and Methods

We used 3.0T MRI scanners (Discovery 750 and Signa Architect, GE Healthcare, Milwaukee, WI, USA) (the patients were fasted from food and water for 6 h before the examination). A conventional axial T1-weighted imaging (T1WI) sequences were acquired with the following parameter settings: repetition time (TR)/echo time (TE) = 450 ms/12 ms, matrix = 512 × 512, field of view (FOV) = 42 cm × 42 cm, section thickness/gap = 3.0 mm/1.0 mm, and number of excitations (NEX) = 2.00. Conventional axial fast-recovery fast spin-echo (FRFSE) T2-weighted imaging (T2WI) sequences were acquired with the following parameter settings: TR/TE = 4000 ms/76 ms, section thickness = 5.0 mm, section gap = 1.0 mm, bandwidth = 85 kHz, matrix = 320 × 320, FOV = 42 cm × 42 cm, and NEX = 2.00. Axial breath-hold IDEAL-IQ sequences were acquired with the following parameter settings: TR = 3.7 ms, TE = 1.7 ms, section thickness = 5.0 mm, bandwidth = 125 kHz, FOV = 42 cm × 42 cm, matrix = 256 × 256, flip angle = 3°, and NEX = 1.00. The following types of images were obtained automatically once the PDFF (IDEAL-IQ sequence) was scanned without off-line processing: in-phase image, out of phase image, pure water image, pure fat image, fat fraction map, and R2* relaxation rate image. The liver fat contents were directly measured using the fat fraction map. Axial breath-hold liver acquisition with volume acceleration (LAVA) sequences were acquired with the following parameter settings: TR = 3.90 ms, TE = 1.80 ms, section thickness = 3.0 mm, section gap = 1.0 mm, matrix = 320 × 160, FOV = 42 cm × 42 cm, and NEX = 1. The contrast agent, gadolinium ethoxybenzyl diethylenetriamine pentaacetic acid, was injected at 2 mL/s, at a dose of 0.2 mmol/kg. MRI scans were performed in four phases, namely the early arterial phase, the late arterial phase, the venous phase, and the equilibrium phase, with delay times of 20 s, 30 s, 65 s, and 240 s, respectively.

All RFA procedures were performed under general anesthesia using a commercially available system (Cool-Tip System™, Radionics, Burlington, MA, USA). The 17-gauge needle electrodes with exposed tip lengths of 3 cm (Single) or 2.5 cm (Cluster) were chosen according to the tumor size and location. Ablation was performed under real-time ultrasonography guidance (Vivid 4, GE Healthcare, Milwaukee, WI, USA; or iU22 system, Philips Healthcare, Bothell, WA, USA) as described in a previous study [14].

### 2.3. Image Analysis

Images were analyzed by two abdominal radiologists with rich diagnostic experience (over 10 years) using the AW4.6 imaging workstation (Sun Microsystems, Santa Clara, CA, USA) with the Functool 9.4 software package (GE Healthcare). The radiologists independently determined the fat content and abnormal perfusion foci in the liver for each group on the PDFF images and the dynamic contrast-enhanced images. The fat content was determined by delineating a circular region of interest (ROI) with a diameter of 8 mm^2^ in each liver segment, and the average value was taken after adding up the content of each segment as the fat content of each liver. The location of the ROI was selected to avoid the surgical site of the tumor and the intrahepatic bile ducts and blood vessels. The fat content measured on a PDFF image is the percentage of fat in the liver. The liver fat content is less than 5% in the normal population, 5–14% in patients with mild fatty liver, 14–28% in patients with moderate fatty liver, and greater than 28% in patients with severe fatty liver [10,15]. The 105 patients with fatty liver were divided into a mild fatty liver group, a moderate fatty liver group, and a severe fatty liver group according to the fat content in the liver. In contrast-enhanced scans, hyperintense signals in the arterial phase and isointense signals in the portal venous and venous phase [16,17] indicate hyperperfusion, while hypointense signals in the arterial phase and isointense signals in the portal venous and venous phase indicate hypoperfusion [18]. Additionally, the number of perfusion abnormalities in each segment of the liver was also analyzed.

### 2.4. Statistical Analysis

Statistical analysis was performed using SPSS 21.0 (Chicago, IL, USA). All data are expressed as the mean ± standard deviation (x ± s). The test level was *α* = 0.05, and *p* < 0.05 was considered statistically significant. Bonferroni’s test was used to compare the liver fat content of the nonfatty liver group with that of each subgroup of the fatty liver group and compare the liver fat content of the same patient before and after reexamination. The correlation between fat content and perfusion disorder was analyzed by logistic regression and expressed by odds ratios (OR). The Bland–Altman plot was used to evaluate the interobserver consistency of the liver fat contents independently measured by the two radiologists [19]. The Kappa coefficient was used to evaluate the interobserver consistency of the liver perfusion disorder.

## 3. Results

The images of all patients were clear and usable. There was no difference in liver fat content between all patients before RFA and the first postoperative MRI examination. In the first postoperative MRI examination, there were five (5/45, 11.11%) patients with hyperperfused foci and three (3/45, 6.67%) patients with hypoperfused foci in the nonfatty liver group; among the 105 patients in the fatty liver group, there were 40 patients in the mild fatty liver group, 35 patients in the moderate fatty liver group and 30 patients in the severe fatty liver group.

**Figure 2:**
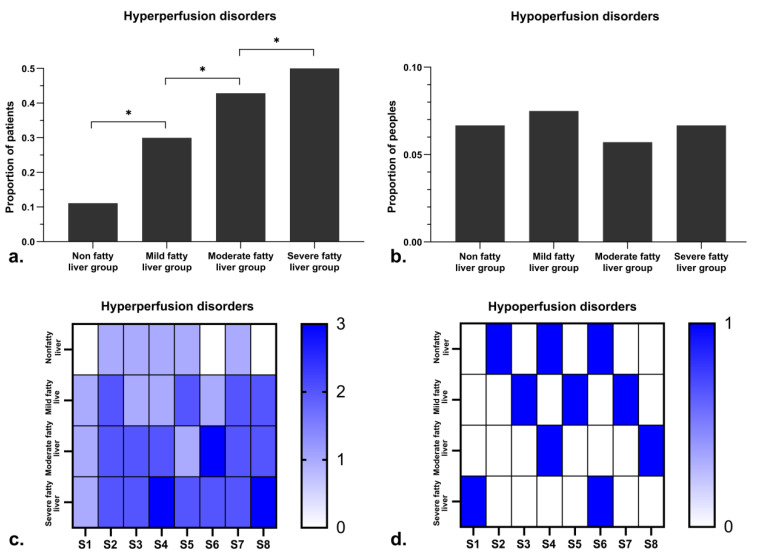
The proportion and number of abnormal perfusion foci in each group of patients and each liver segment. The proportions of patients with hyperperfused foci were significantly different between the nonfatty liver group and the fatty liver group (**a**), whereas the proportions of patients with hypoperfused foci were not significantly different between the nonfatty liver group and the fatty liver group (**b**). The number and distribution of hyperperfusion abnormalities (**c**) and hypoperfusion abnormalities (**d**) in each liver segment were not specific, and there was no statistical difference.

There were 12 (12/40, 30.00%), 15 (15/35, 42.86%), and 17 (17/30, 56.67%) patients with hyperperfused foci in the mild, moderate, and severe fatty liver groups, respectively. The proportions of patients with hyperperfused foci were significantly different between the nonfatty liver group and each subgroup of the fatty liver group (Figure 2a, *p* < 0.05).

Furthermore, there were three (3/40, 7.50%), two (2/35, 5.71%), and two (2/30, 6.67%) patients with hypoperfused foci in the mild, moderate, and severe fatty liver groups, respectively. The proportions of patients with hypoperfused foci were not significantly different between the nonfatty and fatty liver groups (Figure 2b, *p* > 0.05).

The number and distribution of hyperperfusion abnormalities and hypoperfusion abnormalities in each liver segment was not specific, and there was no statistical difference (Figure 2c,d, *p* > 0.05).

In the nonfatty liver group, the liver fat content did not promote hyperperfusion (OR = 0.810) or hypoperfusion abnormalities (OR = 0.328). In the mild, moderate, and severe fatty liver groups, the liver fat content was correlated with hyperperfusion abnormalities (OR = 1.745, OR = 1.446, OR = 1.256, respectively) but was not correlated with hypoperfusion abnormalities (OR = 0.649, OR = 0.798, OR = 0.949, respectively) (Table 2).

**Figure 3:**
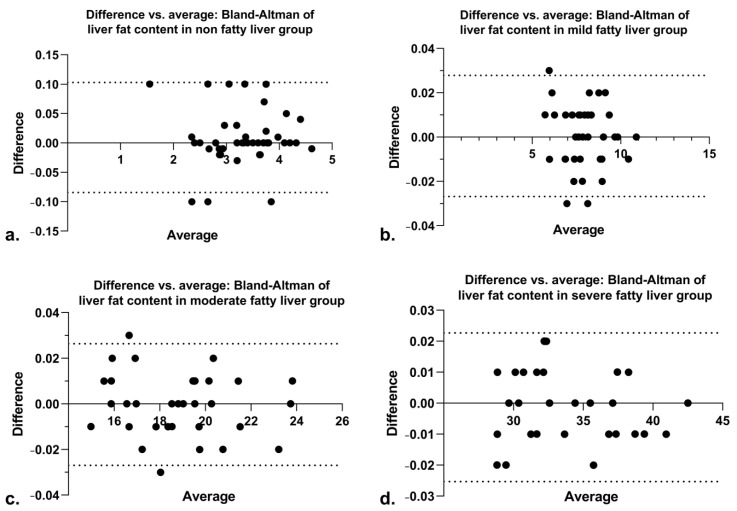
A Bland–Altman plot of the interobserver consistency of the two radiologists’ independent measurements of the liver fat content of the non-(**a**), mild(**b**), moderate(**c**), and severe(**d**) fatty liver groups. The 95% limits of agreement for the four groups were −0.08433 to 0.103, −0.02684 to 0.02784, −0.02696 to 0.02639, and −0.02533 to 0.02266, respectively, indicating high interobserver consistency.

A Bland–Altman plot (Figure 3) was used to analyze the interobserver consistency of the two radiologists’ independent measurements of the liver fat content in the nonfatty liver group, the mild fatty liver group, the moderate fatty liver group, and the severe fatty liver group. The 95% limits of agreement were −0.08433 to 0.103, −0.02684 to 0.02784, −0.02696 to 0.02639, and −0.02533 to 0.02266, respectively, indicating high interobserver consistency. The Kappa coefficients of the liver perfusion disorder in the nonfatty liver group, the mild fatty liver group, the moderate fatty liver group, and the severe fatty liver group were 0.826, 0.835, 0.829, and 0.831, respectively, and it also indicates high interobserver consistency.

**Figure 4:**
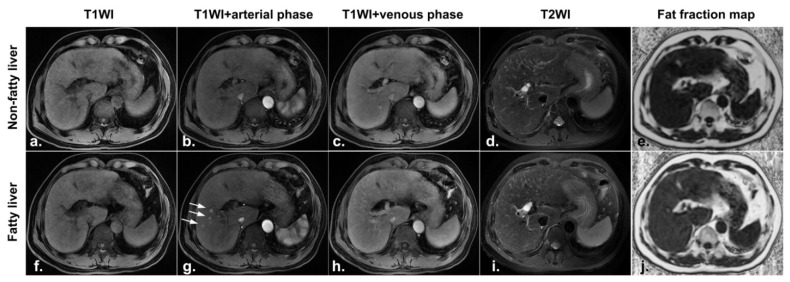
A 51-year-old male patient underwent surgical resection of a small hepatocellular carcinoma. MRI images one month after radiofrequency ablation (**a**–**e**): no lesions were found on both T1W (**a**,**f**) and T2W (**d**,**i**); the fat fraction map showing no fat deposition in the liver (**e**); contrast-enhanced images showing no abnormal perfusion foci (**b**,**c**). MRI images four months after radiofrequency ablation (**f**–**j**): a fat fraction map showing new fat deposition in the liver (**j**); a contrast-enhanced image in the arterial phase showing hyperperfused foci(white arrows) in the liver (**g**); isointense signals in the portal venous phase (**h**).

**Figure 5:**
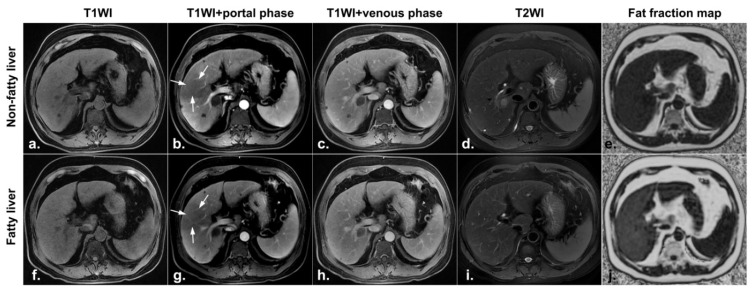
A 46-year-old male patient underwent interventional ablation of a small hepatocellular carcinoma. MRI images one month after radiofrequency ablation (**a**–**e**): no lesions were found on both T1W (**a**,**f**) and T2W (**d**,**i**); the fat fraction map showing no fat deposition in the liver (**e**); a contrast-enhanced image in the arterial phase showing hypoperfused foci(white arrows) in the liver (**b**); isointense signals in the portal venous phase (**c**). MRI images four months after radiofrequency ablation (**f**–**j**): a fat fraction map showing new fat deposition in the liver (**j**); a contrast-enhanced image in the arterial phase showing no obvious change in hypoperfused foci (white arrows) (**g**).

At the second postoperative MRI examination, six patients in the nonfatty liver group were diagnosed with fatty liver, including two patients with newly developed hyperperfusion abnormalities (Figure 4) and one patient whose hypoperfusion abnormality remained the same as it was at the first postoperative MRI examination (Figure 5); the other 39 patients were still free of fatty liver and had no change in HPD.

In the fatty liver group, the liver fat content did not change significantly in any of the patients. There was no change in the hyperperfused or hypoperfused foci when the second postoperative MRI examination.

## 4. Discussion

The involved liver site by HPD appears as an area of hyperintense signal or hypointense signal on the hepatic arterial phase image and returns to a normal signal on the portal venous phase image, which is the same as in other studies [18,20]. HPD are caused by a variety of reasons, and the area of HPD is varied. Radiologists need to be aware of HPD and avoid confusing benign HPD with malignant tumors, resulting in a false positive diagnosis or overestimation of the size of liver tumors [2]. The results of this study show that fatty liver and the liver fat content in postoperative patients with liver cancer were associated with HPD. The higher the liver fat content was, the higher was the risk of hyperperfused foci during the contrast-enhanced MRI scan, whereas the increase in liver fat content did not induce or alter hypoperfused foci.

As a result of in-depth studies on HPD, tumors, trauma, and inflammation have been found to be the leading causes of HPD [16,21,22], while abnormal liver fat deposition may also lead to HPD [5]. However, these studies did not analyze the correlation between liver fat content and the occurrence of HPD. In this study, the PDFF was applied to quantitatively measure the liver fat content of patients after RFA for liver cancer. The results confirmed that the increase in liver fat content led to an increase in hyperperfused foci but barely affected hypoperfused foci. The severity of fatty liver mainly affected hyperperfused foci. The possible mechanism underlying hyperperfusion abnormalities is the compensatory increase in the hepatic artery blood supply due to reduced flow in the portal vein caused by the deposition of fat in the vicinity of the portal vein, which has lower pressure than the hepatic artery [17,18,23]. The possible mechanism underlying hypoperfusion abnormalities is reduced blood supply from the hepatic artery due to its compression by fat deposited in the vicinity of the hepatic artery. In this situation, when the amount of liver fat increases further and compresses the portal vein, the compensatory ability of the hepatic artery is low because it has been oppressed by liver fat, and therefore, there is little change in the hypoperfused foci [24]. However, the patient in this study did not take pathology to analyze the relationship between fat deposition and portal vein, and we plan to use animal models to analyze the relationship in the future. In each group of patients, the distribution of perfusion abnormalities in each liver segment was not specific, and it was speculated that the different microcirculation of each liver segment has little effect on HPD. Therefore, the incidence of perfusion abnormalities was different in patients with different degrees of liver fat content.

The PDFF used in this study can generate six sets of images, including the fat fraction map, through one scan [12,13]. In the past, conventional ultrasound, CT, and MRI examinations could only qualitatively or semi-quantitatively diagnose fat deposition. PDFF can qualitatively diagnose fat deposition and quantitatively measure the degree of fat deposition to provide reliable whole-liver fat content data [11,25], thus providing an accurate analysis of the correlation between liver fat content and abnormal perfusion foci. In the PDFF image, the local fat content of any ROI at any position of the liver can be directly read. This measurement method is simple, fast, accurate, and reproducible, which is consistent with other studies [9]. This clarification of the correlation between the degree of liver fat deposition and the severity of HPD in patients with fatty liver provides a theoretical basis and practical experience for determining whether enhanced foci in the fatty liver after RFA are recurrent tumors or abnormal perfusions, which is of great significance for postoperative review of patients with liver cancer combined with fatty liver.

This study has the following limitations. First, the sample size was small, and the follow-up time was short. In future studies, we will continue to increase the sample size and follow-up time. In addition, patients after RFA did not have CT reexamination, which is related to the hospital’s diagnosis and treatment requirements. In subsequent studies, we will form a multicenter study to include patients with CT reexamination to further investigate whether the procedures impact HPD.

## 5. Conclusions

This study suggested that HPD in the fatty liver can reliably be detected with contrast-enhanced MRI. It confirmed the high correlation between fat content and hyperperfusion abnormalities in the liver after RFA for liver cancer. The higher the postoperative liver fat content was, the higher was the risk of hyperperfusion abnormalities. However, there was little correlation between liver fat content and hypoperfusion abnormalities, and increases in the amount of postoperative liver fat did not induce or change hypoperfused foci. The incidence of perfusion abnormalities is different in patients with different degrees of liver fat content and the different microcirculation of each liver segment have little effect on HPD. The correlation between the degree of liver fat deposition and the severity of HPD provides a theoretical basis and practical experience for determining whether enhanced foci in the fatty liver after RFA are recurrent tumors or abnormal perfusions.

## Figures and Tables

**Table 1:** Clinical data of all patients.

Group	Number	Sex		Mean Age	Hepatitis Virus Infection		Number of Liver Cancer in Each Liver Segment								Alpha-Fetoprotein Levels (ng/mL)	
		Male	Female		HBV	HCB	S1	S2	S3	S4	S5	S6	S7	S8	Preoperative	Postoperative
Nonfatty liver	45	25	20	51.5	44	1	1	7	5	8	3	6	7	8	532.36–936.84	1.21–7.55
Mild fatty liver	40	20	20	51.0	40	0	1	6	5	6	4	5	6	7	594.05–914.95	1.17–7.32
Moderate fatty liver	35	25	10	50.5	34	1	0	5	4	6	3	5	5	7	483.38–969.37	1.67–7.19
Severe fatty liver	30	20	10	49.5	30	0	0	5	4	5	3	4	4	5	492.76–991.65	1.52–7.23

HBV: hepatitis B virus; HCV: hepatitis C virus; S: Liver segment.

**Table 2:** Correlation between liver fat content and hepatic perfusion disorders in the nonfatty liver group and each subgroup of the fatty liver group.

Group	Hyperperfusion Abnormalities			Hypoperfusion Abnormalities		
	B	OR	95% CI for OR	B	OR	95% CI for OR
Nonfatty	−0.211	0.810	0.187 to 3.799	−1.115	0.328	0.043 to 2.039
Mild fatty	0.557	1.745	0.975 to 3.447	−0.432	0.649	0.199 to 1.741
Moderate fatty	0.369	1.446	1.046 to 2.153	−0.226	0.798	0.337 to 1.518
Severe fatty	0.228	1.256	1.017 to 1.635	−0.052	0.949	0.571 to 1.371

B: best-fit values; OR: odds ratios; CI: confidence interval.

## Data Availability

The study did not report any data.
